# MicroRNAs as Potential Regulators of GSK-3β in Renal Cell Carcinoma

**DOI:** 10.3390/cimb45090470

**Published:** 2023-09-11

**Authors:** Masaki Murata, Vladimir Bilim, Yuko Shirono, Akira Kazama, Kaede Hiruma, Masayuki Tasaki, Yoshihiko Tomita

**Affiliations:** 1 Department of Urology, Division of Molecular Oncology, Graduate School of Medical and Dental Sciences, Niigata University, Niigata 951-8510, Japan; vbilim@zoho.com (V.B.); yuko-shirono@med.niigata-u.ac.jp (Y.S.); exfeel@live.jp (A.K.); maple@med.niigata-u.ac.jp (K.H.); masa1214@med.niigata-u.ac.jp (M.T.); niigata-uro@med.niigata-u.ac.jp (Y.T.); 2 Department of Urology, Kameda Daiichi Hospital, Niigata 950-0165, Japan; 3 Glickman Urological and Kidney Institute, Cleveland Clinic, Cleveland, OH 44195, USA

**Keywords:** renal cell carcinoma, competing endogenous RNA, microRNA, hsa-miR-4465, GSK-3β

## Abstract

The prognosis of patients with advanced renal cell carcinoma (RCC) has improved with newer therapies, including molecular-targeted therapies and immuno-oncology agents. Despite these therapeutic advances, many patients with metastatic disease remain uncured. Inhibition of glycogen synthase kinase-3β (GSK-3β) is a promising new therapeutic strategy for RCC; however, the precise regulatory mechanism has not yet been fully elucidated. MicroRNAs (miRNAs) act as post-translational regulators of target genes, and we investigated the potential regulation of miRNAs on GSK-3β in RCC. We selected nine candidate miRNAs from three databases that could potentially regulate GSK-3β. Among these, hsa-miR-4465 (miR-4465) was downregulated in RCC cell lines and renal cancer tissues. Furthermore, luciferase assays revealed that miR-4465 directly interacted with the 3′ untranslated region of GSK-3β, and Western blot analysis showed that overexpression of miR-4465 significantly decreased GSK-3β protein expression. Functional assays showed that miR-4465 overexpression significantly suppressed cell invasion of A498 and Caki-1 cells; however, cell proliferation and migration were suppressed only in Caki-1 and A498 cells, respectively, with no effect on cell cycle and apoptosis. In conclusion, miR-4465 regulates GSK-3β expression but does not consistently affect RCC cell function as a single molecule. Further comprehensive investigation of regulatory networks is required in this field.

## 1. Introduction

Renal cell carcinoma (RCC) is one of the most common cancers, accounting for 2–3% of all carcinomas worldwide, affecting a wide range of ages [1]. While most patients with RCC diagnosed at an early stage can be cured by surgical resection, approximately 30% of patients have metastasis at the time of diagnosis or develop metastasis after curative treatment attempts and require systemic therapy [2]. RCC is well known for its resistance to chemotherapy, and this has often been a challenge in the systemic therapy of metastatic renal cell carcinoma (mRCC); however, the prognosis of patients with mRCC has improved with newer therapies, including molecular-targeted therapy (TT) and immuno-oncology (IO) agents. Several IO and TT combination or IO combination therapy regimens have been approved following pivotal phase III trials, which reported a significant overall survival benefit (37.7 months—not reached) [3,4,5,6,7,8,9]. Despite these therapeutic advances, many patients with metastatic disease remain uncured and combination therapy regimens have been reported to increase various treatment-related adverse events, including cardiovascular adverse events [10,11]. This highlights the need to develop strategies for early diagnosis, identification of patients with a poor prognosis, and novel clinical interventions. 

MicroRNAs (miRNAs) are a family of small noncoding RNAs 19–25 nucleotides in length [12]. Genomic regions capable of generating functional miRNAs are present in diverse locations within the genome, and it is estimated that approximately 50% of miRNAs are expressed in non-protein coding regions [13]. miRNAs are initially generated as long hairpin RNA substrate primary transcripts (pri-miRNAs) by RNA polymerase II [14]. Pri-miRNAs are cleaved by the microprocessor complex Drosha/DiGeorge syndrome critical region gene 8 (DGCR8) into ~70–120 nucleotide miRNA precursors (pre-miRNAs) [15,16]. The processed pre-miRNA is exported to the cytoplasm and cleaved by the RNAse type III enzyme Dicer into an ~18–23 nucleotide mature miRNA duplex [17]. After the double strand is unwound and released as mature single-stranded miRNA, it is incorporated into the RNA-induced silencing complex (RISC) and acts on messenger RNA (mRNA) [18]. Upon binding to the 3′ untranslated region (3′UTR) of mRNAs, mature miRNAs interfere with the translational machinery of their target genes or degrade their target transcripts. Thus, miRNAs act as post-transcriptional regulators of gene expression and are involved in critical biological processes, including development, cell differentiation, proliferation, DNA repair, metabolism, angiogenesis, and apoptosis [19,20,21,22,23,24,25]. Since the first discovery of miRNAs in 1993 [26], numerous new findings have been accumulated from biological studies of miRNAs, including those in the field of cancer [27,28]. Indeed, several miRNAs have been found to have oncogenic or tumor-suppressive functions and are either upregulated or downregulated in tumors. Recently, the utility of non-invasive approaches using free-circulating miRNAs from urine and blood has been reported, and interest in the role of miRNAs as potential biomarkers for early diagnosis, differentiation of benign and malignant tumors, and prognosis prediction in various cancers, including RCC, has increased [29].

Glycogen synthase kinase-3β (GSK-3β) is a widely expressed and highly conserved serine/threonine kinase that phosphorylates various proteins and is involved in several biochemical processes [30]. Many studies have suggested its involvement in various diseases and disorders, including cancer, metabolic disorders, neurological disorders, and immunological disorders [31,32,33,34,35,36,37]. GSK-3β is a positive regulator of nuclear factor kappa beta (NF-κB) activity that increases its downstream anti-apoptotic molecules, B-cell lymphoma factor 2 (Bcl-2) and X-linked inhibitor of apoptosis protein (XIAP), which increase RCC cell survival and chemoresistance [38]. Other reports on RCC indicate that the GSK-3β/4E binding protein 1 (4EBP1) pathway promotes cell proliferation by activating the mammalian target of rapamycin complex 1 (mTORC1) downstream signaling cascade [39]. We have previously identified the antitumor activity of the specific small molecule GSK-3β inhibitor, 9-ING-41, against RCC [40]. Thus, GSK-3β is an important molecule that could potentially be a therapeutic target for RCC; however, its precise regulatory mechanism has not been fully elucidated.

We hypothesized that miRNAs may serve as regulatory factors for GSK-3β expression and cellular function in RCC. Moreover, this phenomenon may be more complex because several factors, including miRNAs, concurrently regulate individual genes, and each miRNA can regulate the expression of multiple targets with complementary sequences to the target region. Currently, the biological significance of most miRNAs remains unknown and requires further functional validation. In this study, we estimated the miRNAs that could regulate GSK-3β expression, analyzed the expression profile of miRNAs in RCC tissues, and investigated their impact on cellular function.

## 2. Materials and Methods

### 2.1. Patient Samples and Clinicopathological Data

Forty-six patients with RCC who received radical nephrectomy and consented to tissue collection between 2016 and 2023 at Niigata University Hospital were included in this study. After excluding five cases of non-clear-cell RCC, 41 patients with clear-cell RCC were included in the final analysis. Renal cancer tissues and paired adjacent normal renal tissues were obtained during the operation and stored in RNAlater Stabilization Solution (Invitrogen; Thermo Fisher Scientific, Inc., Carlsbad, CA, USA) until use. Retrospective longitudinal clinical data were extracted from the hospital’s electronic medical records. This study was conducted in accordance with the Declaration of Helsinki, and the protocol was approved by the Institutional Research Ethics Committee on Genetic Analysis at Niigata University (approval no. G2022-0005). Written informed consent was obtained from all patients before enrolment.

### 2.2. Cell Lines and Cell Culture

In this study, we used human renal cancer cell lines A498, Caki-1, Caki-2, ACHN, KU19-20, KRC/Y, KH39, and a human normal renal proximal tubule epithelial cell line HK-2. A498, Caki-2, ACHN, KRC/Y, KH39, and HK-2 cells were purchased from the American Type Culture Collection (ATCC, Manassas, VA, USA). Caki-1 cells were purchased from the Japanese Collection of Research Bioresources Cell Bank, Osaka, Japan. KU19-20 cells were kindly provided by Dr. Mototsugu Oya (Department of Urology, School of Medicine, Keio University, Tokyo, Japan). All the cell lines were cultured in RPMI 1640 medium (Gibco; Thermo Fisher Scientific, Inc., Grand Island, NY, USA) containing 10% fetal bovine serum (FBS; Gibco; Thermo Fisher Scientific, Inc.) and 90 µg/mL kanamycin at 37 °C in an atmosphere of 5% CO_2_. The cell lines were checked for mycoplasma infection during cell culture.

### 2.3. RNA Isolation and Quantitative Real-Time PCR

Total RNA was extracted from cells or tissues using ISOGEN II (Nippongene, Tokyo, Japan) according to the manufacturer’s instructions. For miRNA assays, TaqMan MicroRNA Assays (Applied Biosystems; Thermo Fisher Scientific, Inc., Carlsbad, CA, USA) were used to quantify the expression levels of mature miRNAs. cDNA was synthesized from total RNA using a TaqMan MicroRNA Reverse Transcription Kit (Applied Biosystems; Thermo Fisher Scientific, Inc.) with assay-specific TaqMan primers, and quantitative real-time PCR was performed using 2× TaqMan Universal PCR Master Mix (Applied Biosystems; Thermo Fisher Scientific, Inc.). The following assay IDs were used: hsa-miR-4465 (hsa-miR-4465, 461889_mat), hsa-miR-9-5p (hsa-miR-9, 000583), hsa-miR-29b-3p (hsa-miR-29b, 000413), and U6 (U6 snRNA, 001973) (all purchased from Applied Biosystems; Thermo Fisher Scientific, Inc.). The reactions were performed on a 7500 Real-Time PCR System (Applied Biosystems; Thermo Fisher Scientific, Inc.). U6 small nuclear RNA was used as an endogenous control, and relative expression levels were calculated using the 2^−ΔΔCt^ method.

### 2.4. Immunohistochemistry

**Figure 1:**
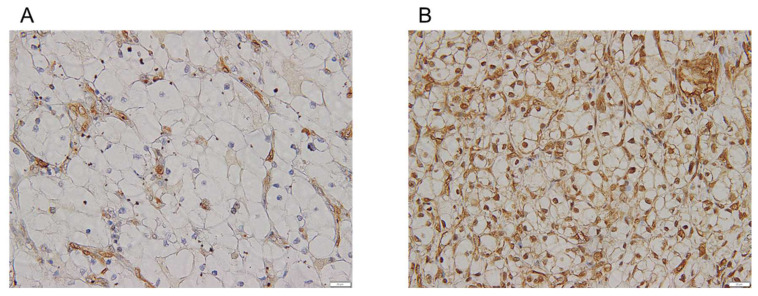
Representative immunohistochemical staining of glycogen synthase kinase-3β (GSK-3β) in tumor tissues obtained from patients with renal cell carcinoma (RCC). (**A**) Negative and (**B**) positive cases. Magnification is ×400. Scale bar = 20 µm.

Immunohistochemical staining was performed using 4 µm thick paraffin sections of RCC tissue specimens. After deparaffinization and rehydration with xylene and ethanol, the epitopes were reactivated by autoclaving (121 °C, 20 min) in 10 mM citrate buffer (pH 6.0). Slides were incubated overnight at 4 °C with the primary antibody and treated with Histofine Simple Stain MAX-PO (Nichirei, Tokyo, Japan) for 30 min at room temperature. The primary antibodies used in this study are listed in Table 1. The staining reaction was developed using DAB in the presence of H2O2, and nuclear counterstaining was performed with Mayer-Hematoxylin Solution (Fujifilm WAKO, Osaka, Japan). The results were observed using an Olympus BX53 microscope (Olympus, Tokyo, Japan). All slides were evaluated for immunostaining without any knowledge of clinical data. Nuclear accumulation of GSK-3β was defined as positive staining of at least 10% of cancer cell nuclei throughout the tumor, regardless of cytoplasmic expression. Representative immunohistochemical staining of negative and positive cases are shown in Figure 1A,B.

### 2.5. Cell Transfection

To verify the effect of hsa-miR-4465 (miR-4465), a miR-4465 mimic (miR-4465) and a mimic-scrambled negative control (miR-NC) were purchased from Invitrogen; Thermo Fisher Scientific, Inc. These reagents were transfected into RCC cell lines using Lipofectamine RNAiMAX Reagent (Invitrogen; Thermo Fisher Scientific, Inc.) according to the manufacturer’s instructions. The targeting sequence was as follows: miR-4465 mimic; CUCAAGUAGUCUGACCAGGGGA.

### 2.6. Luciferase Reporter Assay

The binding sites of miR-4465 on the 3′UTR of GSK-3β were predicted using the ENCORI database (https://rnasysu.com/encori/, accessed on 15 February 2022). Wild-type or mutant sequences were cloned into the pmirGLO Dual-Luciferase miRNA Target Expression Vector (Promega, Madison, WI, USA). Wild-type or mutant reporter plasmids and the miR-4465 mimic or NC were co-transfected into cells using Lipofectamine 3000 Reagent (Invitrogen; Thermo Fisher Scientific, Inc.), according to the manufacturer’s instructions. Luciferase activity was measured 48 h post-transfection using the Dual-Glo Luciferase Assay System (Promega) on a GloMax 96 Microplate Luminometer (Promega). The relative firefly luciferase activity was normalized to the corresponding Renilla luciferase activity. 

### 2.7. Western Blot Analysis

Cells were harvested and lysed using a Cell Lysis Buffer (Cell Signaling Technology, Inc., Danvers, MA, USA) containing a Protease Inhibitor Cocktail (Sigma-Aldrich, St. Louis, MO, USA). The cell lysates were incubated on ice for 30 min, then centrifuged for 30 min (15,000× *g*, 4 °C), and the supernatants were collected. The protein concentration was determined using the Bradford method. Protein samples (20 µg) were separated using 10% SDS-PAGE and then transferred to PVDF membranes. After blocking with 10% skimmed milk in TBS, the membranes were incubated overnight at 4 °C with primary antibodies and then with horseradish peroxidase-labeled secondary antibodies for one hour at room temperature. Immunolabelling bands were visualized using the Clarity Max Western ECL Substrate (Bio-Rad Laboratories, Inc., Hercules, CA, USA) and analyzed using Ez-Capture MG (Atto Corporation, Tokyo, Japan). The primary and secondary antibodies used in the experiments are listed in Table 1. β-actin was used as an endogenous control. Densitometric analysis of band intensity to compare protein expression levels was performed using the ImageJ software program (version 1.53 t).

### 2.8. Cell Proliferation Assay

Cell proliferation was evaluated by MTS assay using CellTiter 96^®^AQueous One Solution Cell Proliferation Assay (Promega) with a tetrazolium compound according to the manufacturer’s instructions. Briefly, cells were seeded in a 96-well plate and incubated for 12 h before transfection. At 0, 24, 48, 72, and 96 h after transfection with siRNA or RNA mimics, 20 µL of CellTiter 96^®^ AQueous One Solution Reagent was added to each well and incubated at 37 °C for 2 h. The absorbance was quantified at 490 nm using an IMARK microplate reader (Bio-Rad Laboratories, Inc.).

### 2.9. Cell Migration Assay

Cell migration was assessed using a wound-healing scratch assay. Cells were seeded into six-well plates. After 12 h, cells were transfected with the desired siRNA using Lipofectamine RNAiMAX. After reaching full confluence, a horizontal line was made using a sterile 1000 µL micropipette tip on the cell culture plates. Wound healing was monitored and imaged at 0, 12, and 24 h post-scratch using an Olympus IX71 microscope (Olympus, Japan). The percent closure was calculated using the following formula:

$$Percent\;Closure\;\left(\%\right)=\frac{A\left(0\right)-A\left(t\right)}{A\left(0\right)}\times 100$$

*A*(0) = area at time zero (0), *A*(t) = area after incubation time.

### 2.10. Transwell Cell Invasion Assay

Cell Invasion was assessed using a CytoSelect 24-well Cell Invasion Assay Kit (Cell Biolabs, Inc., San Diego, CA, USA) with an 8 µm pore size polycarbonate membrane, according to the manufacturer’s instructions. Briefly, after starvation for 24 h in serum-free media, 3.0 × 10^5^ cells that were re-suspended in serum-free media were seeded in the upper chamber. The lower chamber was filled with 500 µL RPMI 1640 medium containing 10% FBS. The plate was incubated at 37 °C in 5% CO_2_ for 24 h to induce the invasion towards the lower chamber. After removing non-invasive cells from the upper side of the chamber, invaded cells on the reverse side were stained using the provided staining solution. The stained membranes were incubated for 10 minutes with extraction buffer and the lysate was quantified by measuring the absorbance at 560 nm using an IMARK microplate reader (Bio-Rad Laboratories, Inc.).

### 2.11. Flow Cytometry Analysis

For cell cycle assessment, cells were harvested 24, 48, and 72 h after transfection. Cells were washed twice with cold PBS and fixed in 70% ethanol at 4 °C for 2 h. After the fixed cells were washed twice with cold PBS, 500 µL of FxCycle™ PI/RNase Staining Solution (Invitrogen; Thermo Fisher Scientific, Inc.) was added and incubated at room temperature in the dark for 20 min. Flow cytometry was performed to assess the cell cycle using a BD FACSCelesta™ Flow Cytometer (BD Biosciences, Franklin Lakes, NJ, USA).

Cell apoptosis was assessed using the MEBCYTO Apoptosis Kit (MBL, Tokyo, Japan) according to the manufacturer’s instructions. Briefly, 48 h after transfection, the cells were washed twice with cold PBS and resuspended in 85 µL Binding Buffer. The suspension was incubated with 10 µL Annexin V-FITC and 5 µL Propidium Iodide (PI) at room temperature in the dark for 15 min. After adding 400 µL of Binding Buffer, cell apoptosis was quantified by flow cytometry using a BD FACSCelesta™ Flow Cytometer (BD Biosciences).

Data analysis was performed using the FlowJo™ software (version 10.9) (BD Biosciences).

### 2.12. Statistical Analysis

The expression status of miRNAs in the RCC cell lines was analyzed using the one-way analysis of variance with Dunnett’s multiple comparison test. Differences in miRNA expression between cancer tissues and matched normal tissues were analyzed using the Wilcoxon matched-pairs signed-rank test. Clinicopathological differences between the two groups were compared using Fisher’s exact test for categorical variables and the Mann–Whitney U test for continuous variables. The Student’s *t*-test was used for comparisons between the two experimental groups. All experimental data were obtained at least in triplicate and are presented as mean ± standard deviation (SD) for the parametric test or median (Interquartile Range (IQR)) for the non-parametric test. Statistical significance was defined as a two-sided *p*-value < 0.05. All statistical analyses were performed using the GraphPad Prism software (version 9.0) (GraphPad Software, Inc., San Diego, CA, USA).

## 3. Results

### 3.1. Estimation of miRNAs Associated with GSK-3β and Their Expression Status in Renal Cell Carcinoma

**Figure 2:**
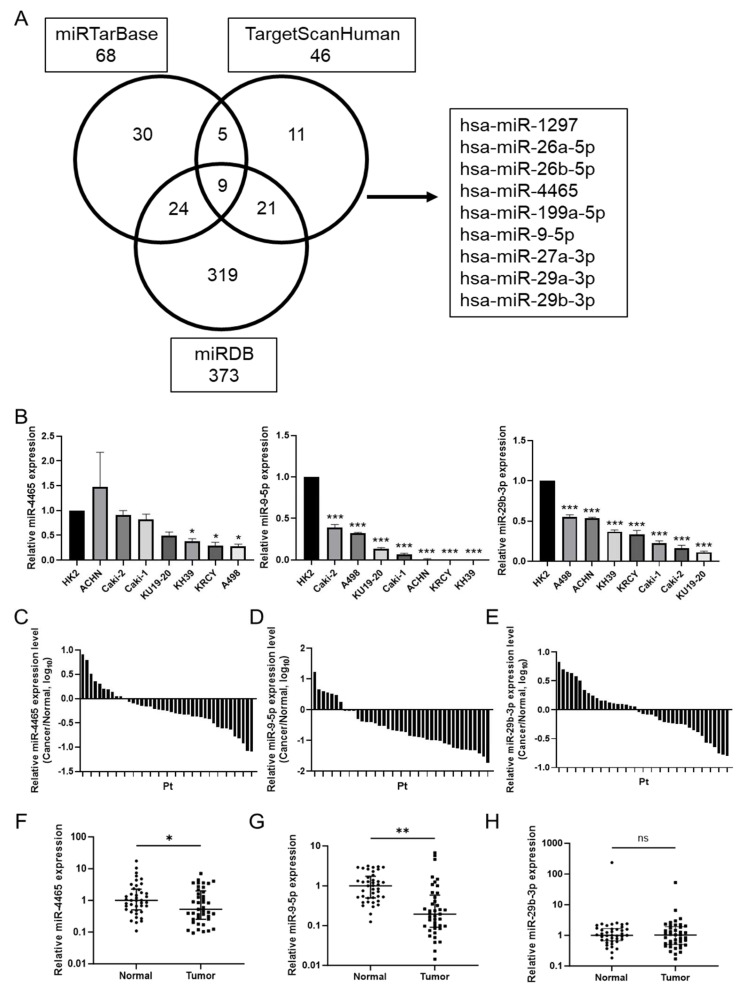
Estimation of MicroRNAs (miRNAs) associated with glycogen synthase kinase-3β (GSK-3β) and their expression status in renal cell carcinoma (RCC). (**A**) Venn diagram showing the candidate miRNAs associated with GSK-3β. Nine miRNAs were predicted to potentially regulate GSK-3β using miRTarBase, TargetScanHuman 8.0, and miRDB. (**B**) The expression levels of the three miRNAs in the normal kidney cell line HK2 and seven RCC cell lines were estimated by RT-qPCR. (**C**–**H**) The expression levels of the three miRNAs in 41 RCC tissues and matched adjacent normal tissues were estimated by RT-qPCR. Pt—patient. Data are shown as mean ± standard deviation (SD) or median ((Interquartile Range (IQR)); ns—not significant; * *p* < 0.05, ** *p* < 0.01, *** *p* < 0.001.

To estimate miRNAs that may regulate GSK-3β, three miRNA databases were used: miRTarBase (https://mirtarbase.cuhk.edu.cn, accessed on 18 October 2021), TargetScanHuman 8.0 (https://www.targetscan.org, accessed on 18 October 2021), and MicroRNA Target Prediction Database [miRDB] (https://mirdb.org, accessed on 18 October 2021). Overlapping miRNAs were extracted from three databases, yielding nine candidate miRNAs (Figure 2A). Among these, hsa-miR-4465 and hsa-miR-29-3p (miR-29b-3p), whose expression has not been studied in RCC, were selected for analysis, and hsa-miR-9-5p (miR-9-5p), which has been previously reported, was analyzed as a reference. 

First, we investigated the expression status of miRNAs in RCC cell lines and samples from patients with RCC using real-time qPCR. Compared to the normal kidney epithelial cell line HK2, the expression of miR-9-5p and miR-29b-3p was significantly decreased in all seven RCC cell lines. The expression of miR-4465 tended to decrease in RCC cell lines compared to that in HK2 cells and was significantly decreased in three RCC cell lines (KH39, KRCY, and A498) (Figure 2B). Compared to matched adjacent normal tissues, miR-4465 (31 of 41 patients, *p* = 0.01) and miR-9-5p (33 of 41 patients, *p* < 0.01) were significantly downregulated in cancer tissues, whereas the difference in miR-29b-3p was not statistically significant (22 of 41 patients, *p* = 0.69) (Figure 2C–H). The relationship between miRNA expression and several clinicopathological characteristics of patients with RCC is shown for each miRNA in Table 2, Table 3 and Table 4. In miR-4465, the TNM stage and histological grade tended to be worse in the upregulation group than in the downregulation group. For miR-9-5p, the M stage was significantly higher in the upregulation group than in the downregulation group; however, there was no correlation between other clinicopathological features and the expression of these three miRNAs. Next, GSK-3β has been shown to accumulate and function in the nucleus of RCC [38], and the relationship between the nuclear accumulation of GSK-3β and several clinicopathological characteristics of patients with RCC was investigated. The results are shown in Table 5. Although not significantly different (possibly due to the small number of patients in this cohort), there was a trend toward a worse histological grade in cases with nuclear accumulation of GSK-3β.

**Table 2:** Association between hsa-miR-4465 expression and clinicopathological characteristics in 41 renal cell carcinoma patients.

	Up-Regulation (*n* = 10)	Down-Regulation (*n* = 31)	*p*-Value
Age, y.o, median (range)	70 (56–77)	74 (49–84)	0.43
Sex, Male/Female (Female: %)	7/3 (30.0%)	20/11 (35.5%)	1.00
Laterality, Left/Right (Right: %)	2/8 (80.0%)	13/18 (58.1%)	0.28
T stage, T1–2/T3–4 (T3–4: %)	2/8 (80.0%)	21/10 (32.3%)	0.01
N stage, N0/N1 (N1: %)	10/0 (0.0%)	31/0 (0.0%)	1.00
M stage, M0/M1 (M1: %)	7/3 (30.0%)	26/5 (16.1%)	0.38
Grade, G1–2/G3–4 (G3–4: %)	3/7 (70.0%)	24/7 (22.6%)	0.02

**Table 3:** Association between hsa-miR-9-5p expression and clinicopathological characteristics in 41 renal cell carcinoma patients.

	Up-Regulation (*n* = 8)	Down-Regulation (*n* = 33)	*p*-Value
Age, y.o, median (range)	67 (58–80)	74 (49–84)	0.08
Sex, Male/Female (Female: %)	5/3 (37.5%)	22/11 (33.3%)	1.00
Laterality, Left/Right (Right: %)	3/5 (62.5%)	12/21 (63.6%)	1.00
T stage, T1–2/T3–4 (T3–4: %)	3/5 (62.5%)	20/13 (39.4%)	0.27
N stage, N0/N1 (N1: %)	8/0 (0.0%)	33/0 (0.0%)	1.00
M stage, M0/M1 (M1: %)	4/4 (50.0%)	29/4 (12.1%)	0.03
Grade, G1–2/G3–4 (G3–4: %)	7/3 (30.0%)	23/13 (36.1%)	0.69

**Table 4:** Association between hsa-miR-29b-3p expression and clinicopathological characteristics in 41 renal cell carcinoma patients.

	Up-Regulation (*n* = 19)	Down-Regulation (*n* = 22)	*p*-Value
Age, y.o, median (range)	68 (31–83)	75 (29–84)	0.07
Sex, Male/Female (Female: %)	11/8 (42.1%)	16/6 (27.3%)	0.35
Laterality, Left/Right (Right: %)	8/11 (57.9%)	7/15 (68.2%)	0.53
T stage, T1–2/T3–4 (T3–4: %)	10/9 (47.4%)	13/9 (40.9%)	0.76
N stage, N0/N1 (N1: %)	19/0 (0.0%)	22/0 (0.0%)	1.00
M stage, M0/M1 (M1: %)	14/5 (26.3%)	19/3 (13.6%)	0.44
Grade, G1–2/G3–4 (G3–4: %)	13/6 (31.6%)	14/8 (36.4%)	1.00

**Table 5:** Association between nuclear glycogen synthase kinase-3β (GSK-3β) staining and clinicopathological characteristics in 41 renal cell carcinoma patients.

	Positive (*n* = 32)	Negative (*n* = 9)	*p*-Value
Age, y.o, median (range)	72 (49–84)	75 (64–83)	0.42
Sex, Male/Female (Female: %)	20/12 (37.5%)	7/2 (22.2%)	0.69
Laterality, Left/Right (Right: %)	12/20 (62.5%)	3/6 (66.7%)	1.00
T stage, T1–2/T3–4 (T3–4: %)	17/15 (46.9%)	6/3 (33.3%)	0.71
N stage, N0/N1 (N1: %)	32/0 (0.0%)	9/0 (0.0%)	1.00
M stage, M0/M1 (M1: %)	26/6 (18.8%)	7/2 (22.2%)	1.00
Grade, G1–2/G3–4 (G3–4: %)	19/13 (40.6%)	8/1 (11.1%)	0.13

### 3.2. hsa-miR-4465 Negatively Regulates the Translation of GSK-3β 

**Figure 3:**
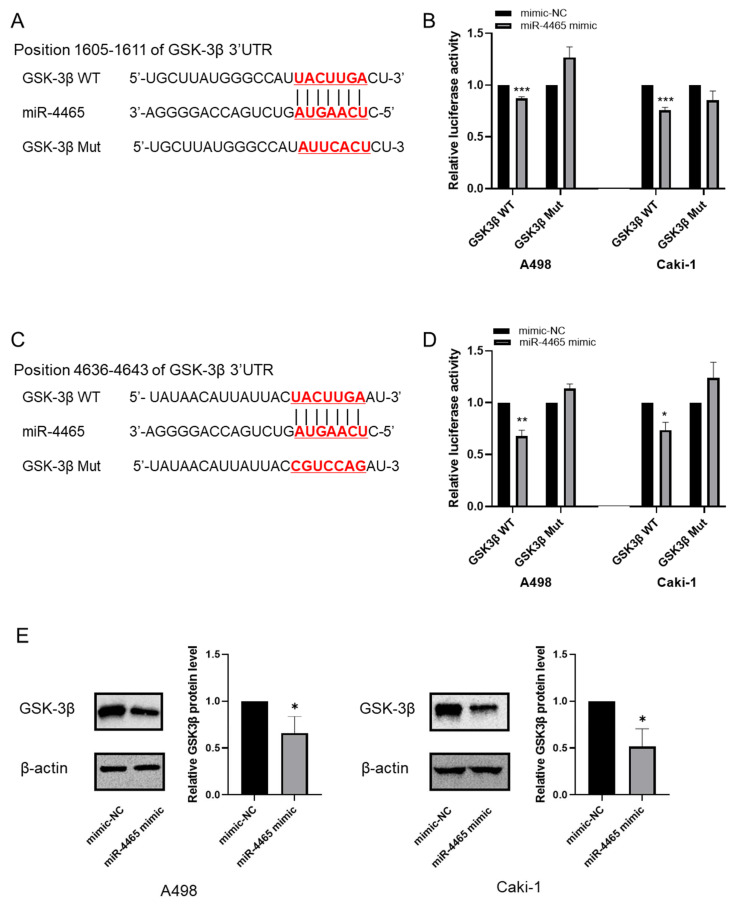
hsa-miR-4465 (miR-4465) deregulates glycogen synthase kinase-3β (GSK-3β) translation in cultured renal cell carcinoma (RCC) cell lines. (**A**,**C**) Binding site of miR-4465 in the 3′ untranslated region (3′UTR) of GSK-3β and the mutant sequence are presented. (**B**,**D**) Luciferase assay detected the luciferase activity of wild-type or mutant GSK-3β–3′UTR co-transfected with miR-4465 mimic or mimic-negative control (mimic-NC). (**E**) Western blot analysis detected the protein levels of GSK-3β after transfection of the cell lines with miR-4465. The intensity of the bands was quantified using the ImageJ software. WT—wild type. Mut—mutant. Data are shown as mean ± standard deviation (SD); * *p* < 0.05, ** *p* < 0.01, *** *p* < 0.001.

We performed further analysis using miR-4465 because it has not been functionally characterized for its role in RCC, and because of its expression status in cell lines and RCC patient samples. Two RCC cell lines (A-498 and Caki-1) with different miR-4465 expression levels were used to evaluate the effects of miR-4465 on cell function. Overexpression of miR-4465 was achieved by transfection of the miR-4465 mimic into A498 and Caki-1 cells, and its expression was increased by 33,162-fold and 1404-fold, respectively. To evaluate whether miR-4465 directly interacts with the 3′UTR of GSK-3β, we performed a luciferase reporter assay. The target sequence was predicted using the TargetScanHuman 8.0 database, and two candidate sequences were detected. For each sequence, a pmirGLO dual-luciferase reporter vector containing the wild-type or mutant GSK-3β–3′UTR was constructed (Figure 3A,C). The results of the luciferase assay validated that co-transfection with wt-GSK-3β and miR-4465 significantly reduced luciferase activity and was more pronounced at positions 4636–4643 (Figure 3B,D). In addition, miR-4465 overexpression significantly reduced the protein expression of GSK-3β in RCC cell lines (Figure 3E). The protein expression levels of XIAP and Bcl-2, which are downstream molecules of GSK-3β, were also reduced. In contrast, miR-4465 did not affect GSK-3α expression (Supplementary Figure S1A,B). Based on these results, miR-4465 was identified as a miRNA that directly targets and regulates GSK-3β via its 3′UTRs.

### 3.3. miR-4465 Differentially Affects Renal Cell Carcinoma Cell Lines

**Figure 4:**
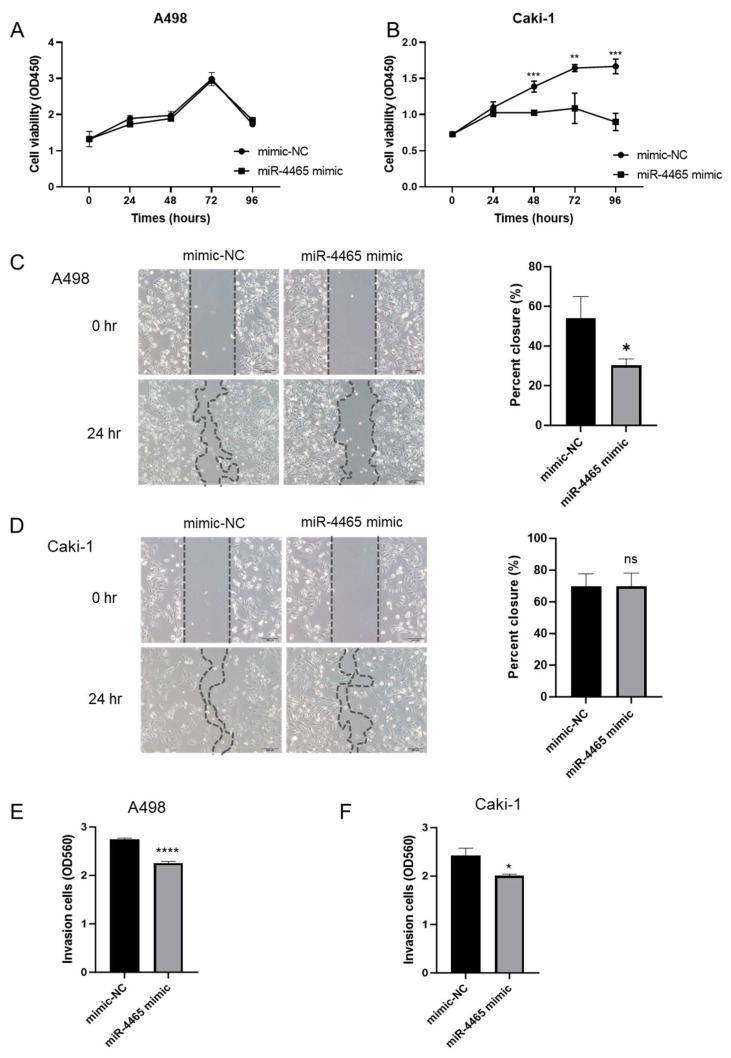
hsa-miR-4465 (miR-4465) differentially affects the viability, migration, and invasion of renal cell carcinoma (RCC) cell lines. (**A**,**B**) Cell proliferation ability of A498 and Caki-1 cells was estimated using MTS assay after overexpression of miR-4465. (**C**,**D**) Cell migration ability of A498 and Caki-1 cells was studied using a scratch assay after overexpression of miR-4465. (**E**,**F**) Cell invasion ability of A498 and Caki-1 cells using a transwell assay after overexpression of miR-4465. OD—optical density. NC—negative control. Data are shown as mean ± standard deviation (SD); ns—not significant, * *p* < 0.05, ** *p* < 0.01, *** *p* < 0.001, **** *p* < 0.0001.

**Figure 5:**
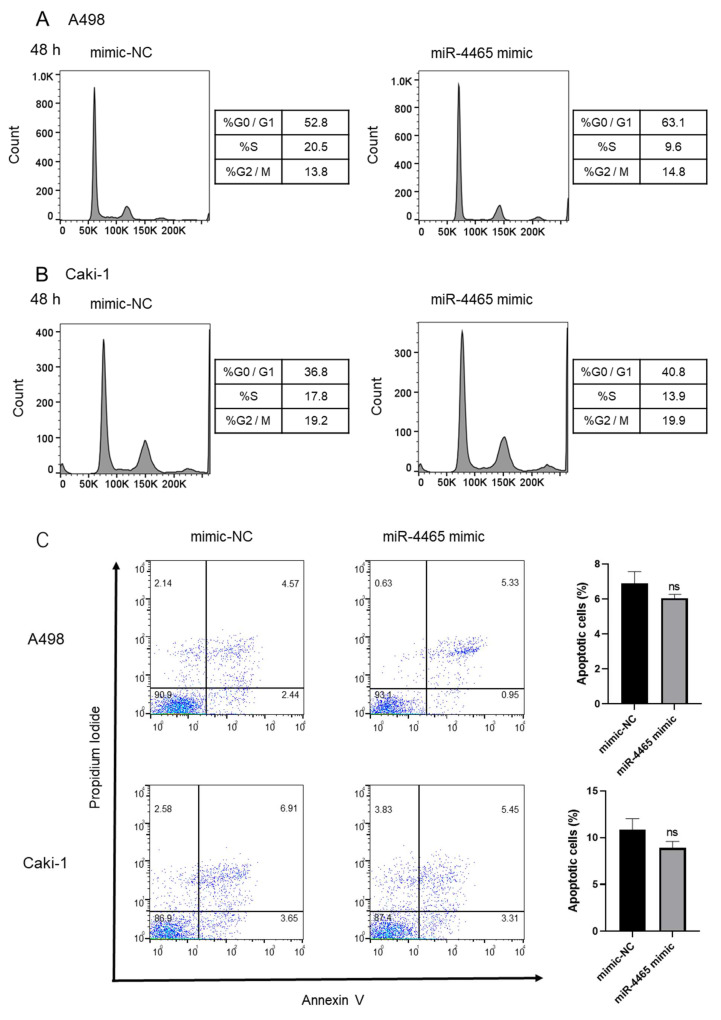
hsa-miR-4465 (miR-4465) does not affect the cell cycle or apoptosis in cultured renal cell carcinoma (RCC) cell lines. (**A**,**B**) Effect of miR-4465 overexpression on the cell cycle of A498 and Caki-1 cells. (**C**) Effect of miR-4465 overexpression on apoptosis of A498 and Caki-1 cells. The cells were stained with Propidium Iodide (PI) or PI and Annexin V and examined using FACScan. Data are shown as mean ± standard deviation (SD); ns—not significant.

To evaluate the biological role of miR-4465 in RCC, we performed functional assays. Overexpression of miR-4465 significantly suppressed cell proliferation in Caki-1 cells (Figure 4B) but had no effect on A498 cells (Figure 4A). In contrast, overexpression of miR-4465 significantly suppressed cell migration in A498 cells (Figure 4C) but did not affect Caki-1 cells (Figure 4D). Cell invasion was significantly inhibited in both A498 and Caki-1 cells (Figure 4E,F). Next, we investigated the cell cycle and cell apoptosis to determine the factors that cause discrepancies in their effects on cell function. However, overexpression of miR-4465 did not affect the cell cycle (Figure 5A,B and Supplementary Figure S2A,B) or apoptosis (Figure 5C). These data suggested that miR-4465 alone had only a partial effect on cellular function in RCC.

## 4. Discussion

Several miRNAs have been reported to play regulatory roles in RCC, and their targets are also diverse [41,42]. Recently, the utility of circulating miRNAs in the peripheral blood of patients has been described, and their application as non-invasive biomarkers for the diagnosis and prediction of therapeutic efficacy has been potentiated [43]. In this context, understanding the underlying regulatory network of miRNAs is important for discussing the mechanisms of renal cancer progression and developing therapeutic strategies.

miR-4465 is a member of the broadly conserved human miR-26 family that shares identical sequences in the seed region. In addition to miR-4465, miR-26a, miR-26b, and miR-1297 belong to this family [44]. Among these, miR-26a/b has been well studied and plays a key role in regulating glucose metabolism, glutamine metabolism, autophagy, and cancer progression in several cell types [45,46,47]. The expression profiles of miR-26 family members in malignant tumors have been described to vary, and miRNA expression may be tissue-specific and influenced by many factors [44]. In fact, miR-4465 has been reported to be involved in several cancers, including hepatocellular carcinoma [48], pancreatic cancer [49], nasopharyngeal carcinoma [50,51], ovarian cancer [52,53], lung cancer [54], and cervical cancer [55]. Out of these, miR-4465 is thought to function as a tumor suppressor except in pancreatic cancer; however, the role of miR-4465 in RCC has not yet been established. We previously reported that treatment with various GSK-3β inhibitors (AR-A014418, SB-216763, TDZD-8, and 9-ING-41) inhibited proliferation and induced cell cycle arrest in RCC cells [38,39,40,56]. Moreover, 9-ING-41 potentiates the antitumor effects of targeted therapeutics and autophagy inhibitors in RCC cells [40]. Based on these results, GSK-3β functions as a tumor promoter in RCC; however, the role of miRNAs in the regulatory mechanism of GSK-3β is unclear. In the present study, we showed that miR-4465 could regulate the expression of GSK-3β by binding directly to its 3′UTR region in RCC but failed to support the role of miR-4465 in RCC progression. 

Post-transcriptional regulation of RNA transcripts by miRNAs functions as competing endogenous RNAs (ceRNAs). That is, there are numerous miRNA-binding sites on RNA transcripts, and all RNA transcripts with miRNA-binding sites form complex gene regulatory networks and regulate each other by specifically competing for shared miRNAs [57]. Multiple targets have been described in miR-4465 [48,49,50,51,52,53,54,55], especially in terms of competitive regulation among RNA transcripts, and miR-4465-mediated changes in gene expression levels and effects on cellular function have been reported among phosphatase and tensine homolog (PTEN), DNA methyltransferase 3β (DNMT3B), and tet methylcytosine dioxygenase 3 (TET3) [58]. It has also been reported that miR-4465 targets PTEN and regulates autophagy by activating the protein kinase B (AKT)/mTOR pathway [59]. Autophagy plays a dichotomous role in cancer, both in inhibiting and promoting cancer growth [60], and its interpretation is complex. Furthermore, activation of the AKT/mTOR pathway is known to play a profound role in cancer progression, and GSK-3β itself is involved in this pathway [61]. Owing to these complex regulatory networks, in addition to regulatory strength, miR-4465 alone may not have had a significant effect on RCC cell function. 

This study observed discrepancies in the functional assays of the two RCC cell lines. Specifically, miR4465 overexpression inhibited the invasion of both A498 and Caki-1 cells, whereas proliferation and migration were inhibited only in A498 and Caki-1 cells, respectively. Cancer cell lines retain unique genetic mutations of the parent tumor from which they are derived, and each has strain-specific morphological features [62]. Moreover, owing to the genetic instability and heterogeneity of original tumors, multiple passages of cell lines result in genetic divergence [63]. Real-world clinical practice has shown that treatment efficacy for RCC varies according to pathological classification and genetic mutation status [64]. We believe that a validation approach that considers gene expression/mutation profiles will be the next challenge in elucidating the molecular mechanisms underlying RCC and developing new therapeutic strategies. We previously confirmed that tumor organoid models can recapitulate RCC’s histological and genetic features [65], and 3D culture (tumor organoids) is considered a better model to overcome these limitations and should be applied in further studies.

Next-generation sequencing technology has enabled the simultaneous detection of multiple genetic mutations. This technology has profoundly impacted our understanding of biological processes in oncology and has led to clinical applications for the personalized treatment of patients based on tumor characteristics [66]. This is no exception in the field of RCC [67]. For further development, a comprehensive understanding of the various underlying processes as well as the genetic phenotype is increasingly required in the era of personalized medicine. Although the effect of each miRNA in regulating the expression of its target gene is not as strong, the resulting network of interactions between downstream effectors could play a crucial role in regulating target gene expression and cellular functions [41]. In the current study, we determined the expression status of three miRNAs potentially related to GSK-3β in RCC using patient samples, and the results showed that miR-4465 and miR-9-5p tended to be downregulated in RCC tissues. However, no correlation was observed among the three miRNAs with worse clinical characteristics, including TNM stage or grade. In addition, since overexpression of miR-4465 negatively regulates GSK-3β expression, we expected it to function as a tumor suppressor in RCC, but on the contrary, worse clinical characteristics were observed in the upregulated group. This observation may result from the effect of miR-4465 on tumorigenesis rather than on tumor progression. We acknowledge that this study has limitations in that the cohort size was small, and diverse clinical stage samples were included. Furthermore, these results have to be interpreted with caution because of the short observation period and lack of data to estimate prognosis. For these reasons, further long-term accumulation of cases and background homogenization are required, which may lead to new findings. Predicting RNA interactions from nucleotide sequences is easy, although it is clear from this study that the actual regulatory ability and potential as a therapeutic target for each carcinoma and its subtypes need to be verified individually. Currently, no miRNA has been widely applied as a biomarker or therapeutic target in clinical practice. It must be emphasized that further knowledge must be accumulated through individual validation, including negative data, to understand the ceRNA network and apply it to therapeutic strategies. 

## Figures and Tables

**Table 1:** Antibodies used in the experiments.

Antibody Name	Product Code	Working Concentration	Manufacture
Western blotting			
GSK-3β Rabbit monoclonal antibody	#12456/D5C5Z	1:1000	Cell Signaling Technology
GSK-3α Rabbit monoclonal antibody	#4337/D80E6	1:1000	Cell Signaling Technology
Purified Mouse Anti-XIAP, clone 28	610716	1:250	BD Transduction Laboratories
BCL2 monoclonal antibody, clone 124	MAB11332	1:200	Abnova, Taipei, Taiwan
β-Actin Mouse monoclonal antibody	#3700/8H10D10	1:1000	Cell Signaling Technology
Anti-Mouse IgG, HRP-Linked Whole Ab Sheep	NA931	1:2500	Cytiva, Tokyo, Japan
Anti-Rabbit IgG, HRP-Linked Whole Ab Donkey	NA934	1:10,000	Cytiva
Immunocytochemistry			
GSK-3β Rabbit monoclonal antibody	#12456/D5C5Z	1:400	Cell Signaling Technology

## Data Availability

The data used and/or analyzed during this study are available from the corresponding author upon reasonable request.

## Supplementary Materials

The following supporting information can be downloaded at: https://www.mdpi.com/article/10.3390/cimb45090470/s1, Figure S1: Protein levels of XIAP, Bcl-2, and GSK-3α in Western blot analysis; Figure S2: Effect of miR-4465 overexpression on the cell cycle 24 and 72 h after transfection.

## References

[1] Sung H., Ferlay J., Siegel R.L., Laversanne M., Soerjomataram I., Jemal A., Bray F. (2021). Global cancer statistics 2020: GLOBOCAN estimates of incidence and mortality worldwide for 36 cancers in 185 countries. CA—A Cancer J. Clin..

[2] Escudier B., Porta C., Schmidinger M., Rioux-Leclercq N., Bex A., Khoo V., Grunwald V., Gillessen S., Horwich A., Comm E.G. (2019). Renal cell carcinoma: ESMO Clinical Practice Guidelines for diagnosis, treatment and follow-up. Ann. Oncol..

[3] Motzer R.J., Tannir N.M., McDermott D.F., Frontera O.A., Melichar B., Choueiri T.K., Plimack E.R., Barthelemy P., Porta C., George S. (2018). Nivolumab plus Ipilimumab versus Sunitinib in Advanced Renal-Cell Carcinoma. N. Engl. J. Med..

[4] Motzer R.J., McDermott D.F., Escudier B., Burotto M., Choueiri T.K., Hammers H.J., Barthelemy P., Plimack E.R., Porta C., George S. (2022). Conditional survival and long-term efficacy with nivolumab plus ipilimumab versus sunitinib in patients with advanced renal cell carcinoma. Cancer.

[5] Rini B.I., Plimack E.R., Stus V., Gafanov R., Hawkins R., Nosov D., Pouliot F., Alekseev B., Soulieres D., Melichar B. (2019). Pembrolizumab plus Axitinib versus Sunitinib for Advanced Renal-Cell Carcinoma. N. Engl. J. Med..

[6] Motzer R.J., Penkov K., Haanen J., Rini B., Albiges L., Campbell M.T., Venugopal B., Kollmannsberger C., Negrier S., Uemura M. (2019). Avelumab plus Axitinib versus Sunitinib for Advanced Renal-Cell Carcinoma. N. Engl. J. Med..

[7] Rini B.I., Powles T., Atkins M.B., Escudier B., McDermott D.F., Suarez C., Bracarda S., Stadler W.M., Donskov F., Lee J.L. (2019). Atezolizumab plus bevacizumab versus sunitinib in patients with previously untreated metastatic renal cell carcinoma (IMmotion151): A multicentre, open-label, phase 3, randomised controlled trial. Lancet.

[8] Choueiri T.K., Powles T., Burotto M., Escudier B., Bourlon M.T., Zurawski B., Juarez V.M.O., Hsieh J.J., Basso U., Shah A.Y. (2021). Nivolumab plus Cabozantinib versus Sunitinib for Advanced Renal-Cell Carcinoma. N. Engl. J. Med..

[9] Motzer R., Alekseev B., Rha S.Y., Porta C., Eto M., Powles T., Grunwald V., Hutson T.E., Kopyltsov E., Mendez-Vidal M.J. (2021). Lenvatinib plus Pembrolizumab or Everolimus for Advanced Renal Cell Carcinoma. N. Engl. J. Med..

[10] Bosma N.A., Warkentin M.T., Gan C.L., Karim S., Heng D.Y.C., Brenner D.R., Lee-Ying R.M. (2022). Efficacy and Safety of First-line Systemic Therapy for Metastatic Renal Cell Carcinoma: A Systematic Review and Network Meta-analysis. Eur. Urol. Open Sci..

[11] Crocetto F., Ferro M., Buonerba C., Bardi L., Dolce P., Scafuri L., Mirto B.F., Verde A., Sciarra A., Barone B. (2023). Comparing cardiovascular adverse events in cancer patients: A meta-analysis of combination therapy with angiogenesis inhibitors and immune checkpoint inhibitors versus angiogenesis inhibitors alone. Crit. Rev. Oncol. Hematol..

[12] Lee L.W., Zhang S.L., Etheridge A., Ma L., Martin D., Galas D., Wang K. (2010). Complexity of the microRNA repertoire revealed by next-generation sequencing. RNA.

[13] Rodriguez A., Griffiths-Jones S., Ashurst J.L., Bradley A. (2004). Identification of mammalian microRNA host genes and transcription units. Genome Res..

[14] Lee Y., Kim M., Han J.J., Yeom K.H., Lee S., Baek S.H., Kim V.N. (2004). MicroRNA genes are transcribed by RNA polymerase II. EMBO J..

[15] Lee Y., Jeon K., Lee J.T., Kim S., Kim V.N. (2002). MicroRNA maturation: Stepwise processing and subcellular localization. EMBO J..

[16] Lee Y., Ahn C., Han J.J., Choi H., Kim J., Yim J., Lee J., Provost P., Radmark O., Kim S. (2003). The nuclear RNase III Drosha initiates microRNA processing. Nature.

[17] Chendrimada T.P., Gregory R.I., Kumaraswamy E., Norman J., Cooch N., Nishikura K., Shiekhattar R. (2005). TRBP recruits the Dicer complex to Ago2 for microRNA processing and gene silencing. Nature.

[18] Schwarz D.S., Hutvagner G., Du T., Xu Z.S., Aronin N., Zamore P.D. (2003). Asymmetry in the assembly of the RNAi enzyme complex. Cell.

[19] Chen C.Z., Li L., Lodish H.F., Bartel D.P. (2004). MicroRNAs modulate hematopoietic lineage differentiation. Science.

[20] Hayashita Y., Osada H., Tatematsu Y., Yamada H., Yanagisawa K., Tomida S., Yatabe Y., Kawahara K., Sekido Y., Takahashi T. (2005). A polycistronic microRNA cluster, miR-17-92, is overexpressed in human lung cancers and enhances cell proliferation. Cancer Res..

[21] Crosby M.E., Kulshreshtha R., Ivan M., Glazer P.M. (2009). MicroRNA regulation of DNA repair gene expression in hypoxic stress. Cancer Res..

[22] Xu P., Vernooy S.Y., Guo M., Hay B.A. (2003). The Drosophila microRNA Mir-14 suppresses cell death and is required for normal fat metabolism. Curr. Biol..

[23] Poliseno L., Tuccoli A., Mariani L., Evangelista M., Citti L., Woods K., Mercatanti A., Hammond S., Rainaldi G. (2006). MicroRNAs modulate the angiogenic properties of HUVECs. Blood.

[24] Calin G.A., Dumitru C.D., Shimizu M., Bichi R., Zupo S., Noch E., Aldler H., Rattan S., Keating M., Rai K. (2002). Frequent deletions and down-regulation of micro- RNA genes miR15 and miR16 at 13q14 in chronic lymphocytic leukemia. Proc. Natl. Acad. Sci. USA.

[25] Lewis B.P., Shih I.H., Jones-Rhoades M.W., Bartel D.P., Burge C.B. (2003). Prediction of mammalian microRNA targets. Cell.

[26] Lee R.C., Feinbaum R.L., Ambros V. (1993). The c-elegans heterochronic gene lin-4 encodes small rnas with antisense complementarity to LIN-14. Cell.

[27] Lu J., Getz G., Miska E.A., Alvarez-Saavedra E., Lamb J., Peck D., Sweet-Cordero A., Ebet B.L., Mak R.H., Ferrando A.A. (2005). MicroRNA expression profiles classify human cancers. Nature.

[28] MacFarlane L.A., Murphy P.R. (2010). MicroRNA: Biogenesis, Function and Role in Cancer. Curr. Genom..

[29] Aveta A., Cilio S., Contieri R., Spena G., Napolitano L., Manfredi C., Franco A., Crocerossa F., Cerrato C., Ferro M. (2023). Urinary MicroRNAs as Biomarkers of Urological Cancers: A Systematic Review. Int. J. Mol. Sci..

[30] Kockeritz L., Doble B., Patel S., Woodgett J.R. (2006). Glycogen synthase kinase-3—An overview of an over-achieving protein kinase. Curr. Drug Targets.

[31] Gao C., Holscher C., Liu Y.Z., Li L. (2012). GSK3: A key target for the development of novel treatments for type 2 diabetes mellitus and Alzheimer disease. Rev. Neurosci..

[32] Amar S., Belmaker R.H., Agam G. (2011). The Possible Involvement of Glycogen Synthase Kinase-3 (GSK-3) in Diabetes, Cancer and Central Nervous System Diseases. Curr. Pharm. Des..

[33] Hur E.M., Zhou F.Q. (2010). GSK3 signalling in neural development. Nat. Rev. Neurosci..

[34] O’Leary O., Nolan Y. (2015). Glycogen synthase kinase-3 as a therapeutic target for cognitive dysfunction in neuropsychiatric disorders. CNS Drugs.

[35] Li Y.C., Gao W.J. (2011). GSK-3β activity and hyperdopamine-dependent behaviors. Neurosci. Biobehav. Rev..

[36] Wang H.Z., Brown J., Martin M. (2011). Glycogen synthase kinase 3: A point of convergence for the host inflammatory response. Cytokine.

[37] Duda P., Akula S.M., Abrams S.L., Steelman L.S., Martelli A.M., Cocco L., Ratti S., Candido S., Libra M., Montalto G. (2020). Targeting GSK3 and Associated Signaling Pathways Involved in Cancer. Cells.

[38] Bilim V., Ougolkov A., Yuuki K., Naito S., Kawazoe H., Muto A., Oya M., Billadeau D., Motoyama T., Tomita Y. (2009). Glycogen synthase kinase-3: A new therapeutic target in renal cell carcinoma. Br. J. Cancer.

[39] Ito H., Ichiyanagi O., Naito S., Bilim V.N., Tomita Y., Kato T., Nagaoka A., Tsuchiya N. (2016). GSK-3 directly regulates phospho-4EBP1 in renal cell carcinoma cell-line: An intrinsic subcellular mechanism for resistance to mTORC1 inhibition. BMC Cancer.

[40] Anraku T., Kuroki H., Kazama A., Bilim V., Tasaki M., Schmitt D., Mazar A., Giles F.J., Ugolkov A., Tomita Y. (2020). Clinically relevant GSK-3β inhibitor 9-ING-41 is active as a single agent and in combination with other antitumor therapies in human renal cancer. Int. J. Mol. Med..

[41] Ghafouri-Fard S., Shirvani-Farsani Z., Branicki W., Taheri M. (2020). MicroRNA Signature in Renal Cell Carcinoma. Front. Oncol..

[42] Guo Y.H., Li X.B., Zheng J.B., Fang J.L., Pan G.H., Chen Z. (2021). Identification of a novel immune-related microRNA prognostic model in clear cell renal cell carcinoma. Transl. Androl. Urol..

[43] Chanudet E., Wozniak M.B., Bouaoun L., Byrnes G., Mukeriya A., Zaridze D., Brennan P., Muller D.C., Scelo G. (2017). Large-scale genome-wide screening of circulating microRNAs in clear cell renal cell carcinoma reveals specific signatures in late stage disease. Int. J. Cancer.

[44] Li C.G., Li Y.Y., Lu Y.F., Niu Z.R., Zhao H.A., Peng Y., Li M.L. (2021). miR-26 family and its target genes in tumorigenesis and development. Crit. Rev. Oncol. Hematol..

[45] Chen B., Liu Y.L., Jin X.W., Lu W.L., Liu J.J., Xia Z.J., Yuan Q., Zhao X., Xu N.Z., Liang S.F. (2014). MicroRNA-26a regulates glucose metabolism by direct targeting PDHX in colorectal cancer cells. BMC Cancer.

[46] Liu X.X., Li X.J., Zhang B., Liang Y.J., Zhou C.X., Cao D.X., He M., Chen G.Q., He J.R., Zhao Q. (2011). MicroRNA-26b is underexpressed in human breast cancer and induces cell apoptosis by targeting SLC7A11. FEBS Lett..

[47] Jin F.F., Wang Y.B., Li M.Z., Zhu Y.A., Liang H.W., Wang C., Wang F., Zhang C.Y., Zen K., Li L.M. (2017). MiR-26 enhances chemosensitivity and promotes apoptosis of hepatocellular carcinoma cells through inhibiting autophagy. Cell Death Dis..

[48] Bu W.J., Fang Z., Li W.L., Wang X., Dong M.J., Tao Q.Y., Zhang L., Xu Y.Q. (2020). LINC00240 sponges miR-4465 to promote proliferation, migration, and invasion of hepatocellular carcinoma cells via HGF/c-MET signaling pathway. Eur. Rev. Med. Pharmacol. Sci..

[49] Cao W.P., Zeng Z.R., He Z.W., Lei S. (2021). Hypoxic pancreatic stellate cell-derived exosomal mirnas promote proliferation and invasion of pancreatic cancer through the PTEN/AKT pathway. Aging.

[50] Li Q.G., Xiao T., Zhu W., Yu Z.Z., Huang X.P., Yi H., Lu S.S., Tang Y.Y., Huang W., Xiao Z.Q. (2020). HDAC7 promotes the oncogenicity of nasopharyngeal carcinoma cells by miR-4465-EphA2 signaling axis. Cell Death Dis..

[51] Zhong Q., Wang Z.Q., Liao X.H., Wu R.R., Guo X.Q. (2020). LncRNA GAS5/miR-4465 axis regulates the malignant potential of nasopharyngeal carcinoma by targeting COX2. Cell Cycle.

[52] Zhao H., Wang A.X., Zhang Z.W. (2020). LncRNA SDHAP1 confers paclitaxel resistance of ovarian cancer by regulating EIF4G2 expression via miR-4465. J. Biochem..

[53] Wu Y. (2019). Long noncoding RNA SNHG6 promotes cell proliferation and migration through sponging mir-4465 in ovarian clear cell carcinoma. Gynecol. Oncol..

[54] Sun J., Tian X., Lu S.Q., Hu H.B. (2017). MicroRNA-4465 suppresses tumor proliferation and metastasis in non-small cell lung cancer by directly targeting the oncogene EZH2. Biomed. Pharmacother..

[55] Tang X., Wen X.M., Li Z.Y., Wen D.X., Lin L., Liu J.Q., Li M.Y. (2021). Hsa_circ_0102171 aggravates the progression of cervical cancer through targeting miR-4465/CREBRF axis. J. Cell. Physiol..

[56] Kawazoe H., Bilim V.N., Ugolkov A.V., Yuuki K., Naito S., Nagaoka A., Kato T., Tomita Y. (2012). GSK-3 inhibition in vitro and in vivo enhances antitumor effect of sorafenib in renal cell carcinoma (RCC). Biochem. Biophys. Res. Commun..

[57] Bai S.H., Wu Y.Y., Yan Y.L., Shao S., Zhang J.Z., Liu J.X., Hui B.N., Liu R., Ma H.L., Zhang X.Z. (2020). Construct a circRNA/miRNA/mRNA regulatory network to explore potential pathogenesis and therapy options of clear cell renal cell carcinoma. Sci. Rep..

[58] Roquid K.A.R., Alcantara K.M.M., Garcia R.L. (2020). Identification and validation of mRNA 3′untranslated regions of DNMT3B and TET3 as novel competing endogenous RNAs of the tumor suppressor PTEN. Int. J. Oncol..

[59] Tao Z.T., Feng C.X., Mao C.M., Ren J., Tai Y.S., Guo H.J., Pu M., Zhou Y., Wang G.H., Wang M. (2019). MiR-4465 directly targets PTEN to inhibit AKT/mTOR pathway-mediated autophagy. Cell Stress Chaperones.

[60] Onorati A.V., Dyczynski M., Ojha R., Amaravadi R.K. (2018). Targeting Autophagy in Cancer. Cancer.

[61] Shaw R.J., Cantley L.C. (2006). Ras, PI(3)K and mTOR signalling controls tumour cell growth. Nature.

[62] Greshock J., Nathanson K., Martin A.M., Zhang L., Coukos G., Weber B.L., Zaks T.Z. (2007). Cancer cell lines as genetic models of their parent histology: Analyses based on array comparative genomic hybridization. Cancer Res..

[63] Ben-David U., Siranosian B., Ha G., Tang H., Oren Y., Hinohara K., Strathdee C.A., Dempster J., Lyons N.J., Burns R. (2018). Genetic and transcriptional evolution alters cancer cell line drug response. Nature.

[64] Yamana K., Ohashi R., Tomita Y. (2022). Contemporary Drug Therapy for Renal Cell Carcinoma-Evidence Accumulation and Histological Implications in Treatment Strategy. Biomedicines.

[65] Kazama A., Anraku T., Kuroki H., Shirono Y., Murata M., Bilim V., Ugolkov A., Saito K., Tomita Y. (2021). Development of patient-derived tumor organoids and a drug testing model for renal cell carcinoma. Oncol. Rep..

[66] Kamps R., Brandao R.D., van den Bosch B.J., Paulussen A.D.C., Xanthoulea S., Blok M.J., Romano A. (2017). Next-Generation Sequencing in Oncology: Genetic Diagnosis, Risk Prediction and Cancer Classification. Int. J. Mol. Sci..

[67] Fiorentino M., Gruppioni E., Massari F., Giunchi F., Altimari A., Ciccarese C., Bimbatti D., Scarpa A., Iacovelli R., Porta C. (2017). Wide spetcrum mutational analysis of metastatic renal cell cancer: A retrospective next generation sequencing approach. Oncotarget.

