# Supplementary material for: MicroRNAs as Potential Regulators of GSK-3β in Renal Cell Carcinoma
Source: Curr Issues Mol Biol. 2023 Sep 11;45(9):7432–48. doi: 10.3390/cimb45090470 (PMC10529713; doi:10.3390/cimb45090470)
Supplement: Supplementary file 1 [file cimb-45-00470-s001.zip › cimb-2512560-supplementary.pdf]

MicroRNAs as Potential Regulators of GSK-3β in Renal Cell Carcinoma

Supplementary Materials

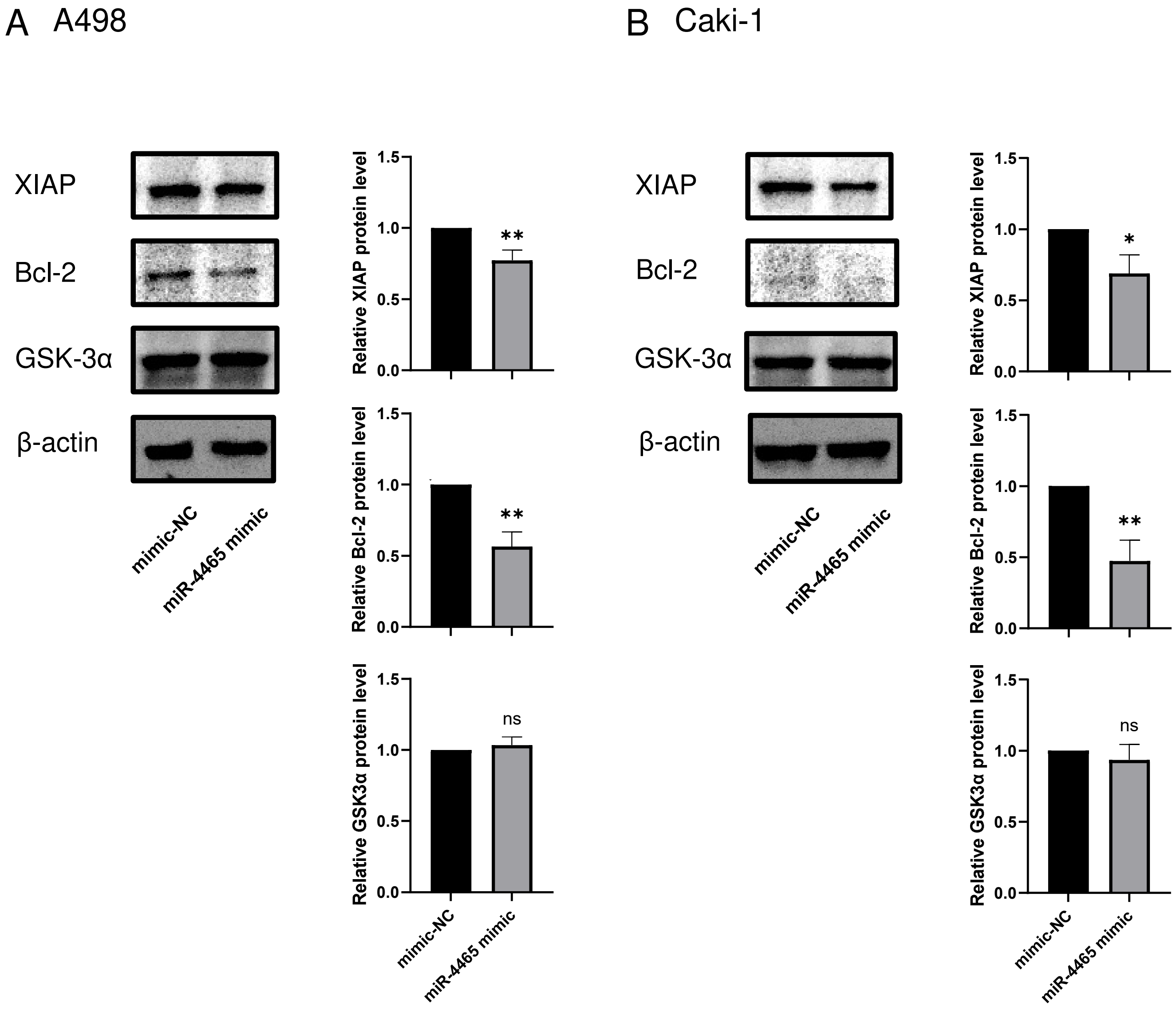

Supplementary Figure 1. (A, B) Western blot analysis detected the protein levels of X-linked inhibitor of apoptosis protein (XIAP), B-cell lymphoma factor 2 (Bcl-2), and glycogen synthase kinase-3α (GSK-3α) after transfection of the cell lines with hsa-miR-4465 (miR-4465). A: A498 cells, B: Caki-1 cells. The intensity of the bands was quantified using the ImageJ software. NC - negative control. Data are shown as mean ± standard deviation (SD); ns - not significant; *p < 0.05, **p < 0.01.

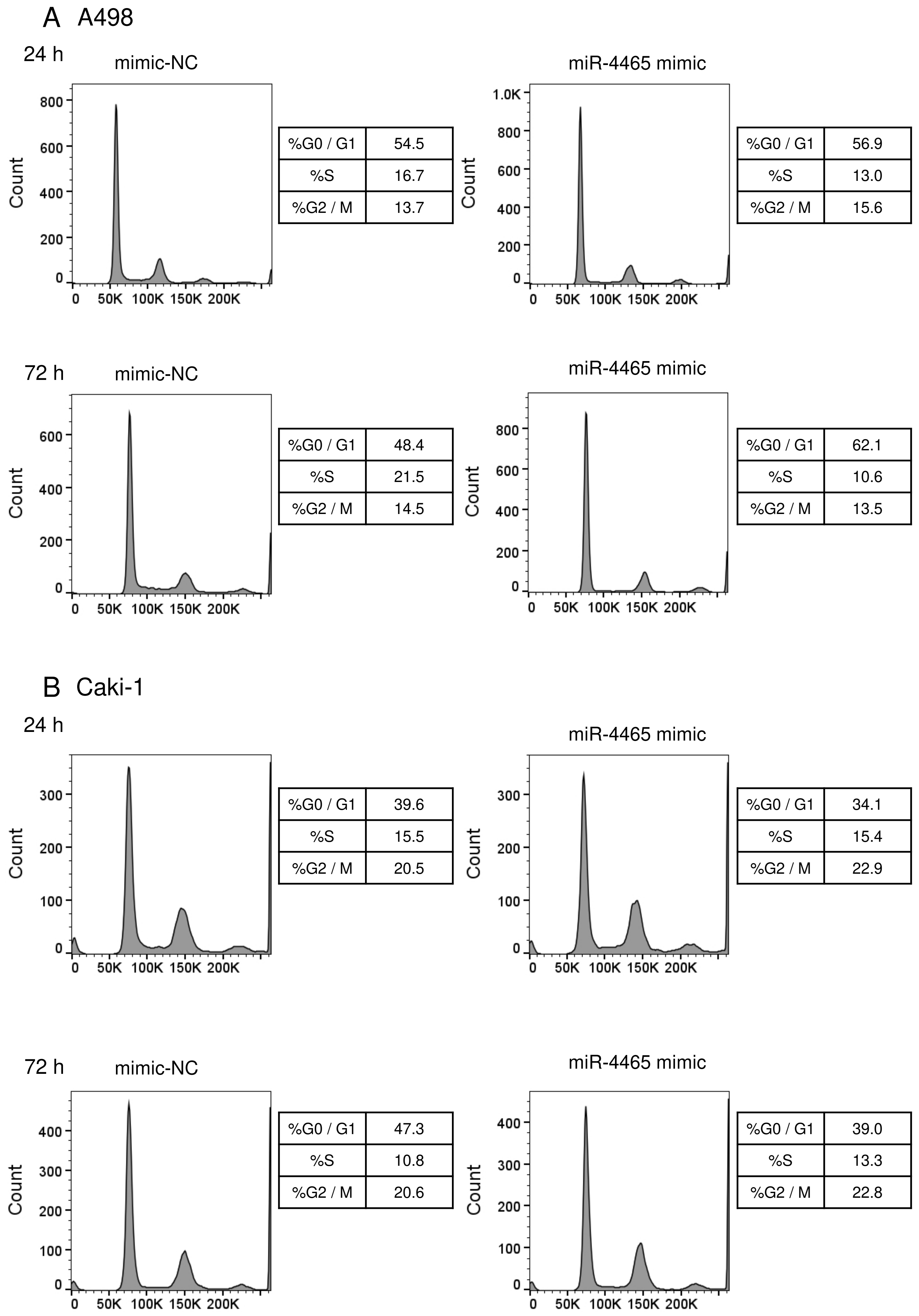

Supplementary Figure 2. (A,B) Effect of hsa-miR-4465 (miR-4465) overexpression on the cell cycle 24 and 72 hours after transfection. A: A498 cells, B: Caki-1 cells.
